# In Vitro and In Vivo Evaluation of Injectable Strontium-Modified Calcium Phosphate Cement for Bone Defect Repair in Rats

**DOI:** 10.3390/ijms24010568

**Published:** 2022-12-29

**Authors:** Hailiang Xu, Lei Zhu, Fang Tian, Chengwen Wang, Weidong Wu, Botao Lu, Liang Yan, Shuaijun Jia, Dingjun Hao

**Affiliations:** 1 Department of Spine Surgery, Honghui Hospital, Xi’an Jiaotong University, Xi’an 710054, China; 2 Shaanxi Key Laboratory of Spine Bionic Treatment, Xi’an 710054, China

**Keywords:** strontium, tristrontium silicate, calcium phosphate cement, osteogenesis, inflammation, mechanical property

## Abstract

Calcium phosphate cement (CPC) has been widely studied, but its lack of osteoinductivity and inadequate mechanical properties limit its application, while strontium is able to promote bone formation and inhibit bone resorption. In this study, different proportions of tristrontium silicate were introduced to create a novel strontium-modified calcium phosphate cement (SMPC). The physicochemical properties of SMPC and CPC were compared, and the microstructures of the bone cements were characterized with scanning electron microscopy assays. Then, the effect of SMPC on cell proliferation and differentiation was examined. Furthermore, local inflammatory response and osteogenesis after SMPC implantation were also confirmed in the study. Finally, a rat model of isolated vertebral defects was used to test the biomechanical properties of the cements. The results showed that SMPC has better injectability and a shorter setting time than CPC. Meanwhile, the addition of tristrontium silicate promoted the mechanical strength of calcium phosphate cement, and the compressive strength of 5% SMPC increased to 6.00 ± 0.74 MPa. However, this promotion effect gradually diminished with an increase in tristrontium silicate, which was also found in the rat model of isolated vertebral defects. Furthermore, SMPC showed a more preferential role in promoting cell proliferation and differentiation compared to CPC. Neither SMPC nor CPC showed significant inflammatory responses in vivo. Histological staining suggested that SMPCs were significantly better than CPC in promoting new bone regeneration. Importantly, this osteogenesis effect of SMPC was positively correlated with the ratio of tristrontium silicate. In conclusion, 5% SMPC is a promising substitute material for bone repair with excellent physicochemical properties and biological activity.

## 1. Introduction

The incidence of osteoporosis is rising as the global population ages, and the most frequent fracture in patients with osteoporosis is an osteoporotic vertebral compression fracture (OVCF), with a reported incidence of 307 cases per 100,000 people [1,2]. Percutaneous kyphoplasty (PKP) and percutaneous vertebroplasty (PVP), which include the local implantation of bone substitutes to restore the spinal sequence and fix fractures, are the most common clinical surgical therapy strategies for OVCF [1,3]. Polymethyl methacrylate (PMMA) is frequently used as an embedding medium [4]. However, there is increasing evidence that the prognosis of OVCF patients is adversely affected by the thermal effects of PMMA curing and its non-degradable characteristics [5,6,7].

Injectable calcium phosphate cement (CPC) is one of the most promising materials for future clinical applications. The mechanical features, functions, and modifications of CPC have been extensively studied since it was invented in 1982 by Brown and Chow [8,9]. Apatite and brushite are the most typical end products of CPC and are quite close to the composition and structure of the mineral phase of bone [10,11,12]. CPC and its produced scaffold materials can serve as a substrate for the attachment and proliferation of osteoblasts and neogenesis of the blood vasculature in bone defects [13]. CPC is often used in the fields of orthopedic surgery, stomatology, and oral and maxillofacial surgery due to its injectability and biodegradability [14,15,16]. However, its lack of osteoinductivity and biomechanical stability restricts its effectiveness [17,18].

Many active ingredients have been incorporated into the composition of CPCs in recent years in an effort to address these drawbacks. Synthetic polymers (polycaprolactone (PCL) and polylactic acid (PLA)) and bone morphogenic proteins (BMP-2, BMP-9, etc.) have been found to enhance the osteoinductive activity and mechanical properties of CPCs [19,20,21]. However, there is evidence that BMPs can cause heterotopic osteogenesis [22,23]. The mechanical characteristics of synthetic polymer materials decline uncontrollably, making it impossible to preserve their mechanical properties after prolonged transplantation in vivo [24]. In addition, adding synthetic polymeric components to CPC did not significantly enhance its osteoinductivity [25,26]. It has been found that some active metal ions can improve the mechanical and biological properties of CPCs. These active metal ions account for less than 0.02% of the total body mass, but they play a critical role in various physiological processes, regulating enzyme activity and modulating cellular functions [27]. Increased bioactivity and shortening of the cement’s setting time are achieved by adding magnesium ions to CPC [28]. In addition to affecting cement rheology and setting time, magnesium-containing calcium phosphate microspheres can also enhance osteogenesis through anti-inflammatory effects [29]. Nevertheless, magnesium metal is highly reactive and tends to produce hydrogen gas in vivo, which limits its application in CPC preparation [30]. Incorporating zinc ions increases CPC’s compressive characteristics, which, in turn, aids in angiogenesis [31]. However, the safe concentration range of zinc ions in the body is very narrow, and it is challenging to precisely control the slow release of zinc in the body without toxicity [32,33]. Finding an appropriate active metal ion for CPC modification is imperative.

Strontium (Sr) has been demonstrated to promote bone formation and inhibit bone resorption, two processes that play out primarily in the skeletal system of the human body [34,35,36]. Strontium can improve the structural strength of bone by increasing the volume of bone trabeculae [37]. Strontium ranelate (Portelos) and other Sr-containing medications have been recommended for the treatment of osteoporosis. While the systemic application of Sr-containing drugs has several adverse effects, the topical delayed release of strontium is a risk-free therapy approach [38]. Many researchers have attempted to improve CPC’s bioactivity by incorporating Sr. SrHPO_4_, SrCO_3_, etc. were reported to introduce bioactive strontium ions into CPCs, and the bioactivity and mechanical properties of CPCs were improved to varying degrees [34,39]. There is a dose–response relationship between strontium and bone formation [40]. The type of Sr-containing compounds and the proportion of Sr significantly impact the biological activity and mechanical properties of CPCs, which determines the structure and physicochemical properties of the end product [34,39,40]. Tristrontium silicate (Sr_3_SiO_5_) introduces bioactive Sr ions and a Sr concentration of 0.75/mol. Since the strontium content of tristrontium silicate is relatively high, its incorporation into CPC ensures the strontium content without reducing the proportion of inorganic calcium elements. Furthermore, the optimal incorporation ratio of tristrontium silicate in CPC has not been reported.

This study is significant because we created a novel strontium-modified calcium phosphate cement (SMPC), with different proportions of tristrontium silicate, which could release bioactive strontium ions sustainably and stimulate new bone formation. We characterized the microstructure of the SMPCs using scanning electron microscopy assays. Meanwhile, the cytotoxic and osteoinductive properties of SMPCs were examined by culture together with osteoblasts. We also investigated the effects of different ratios of tristrontium silicate on CPC’s mechanical properties and osteoblasts’ biological functions. The local inflammatory effects of SMPC were also verified in the subcutaneous implantation model study. The femoral condylar defect implantation model was used to evaluate the osteogenic capacity of bone cement in vivo. Finally, a rat model of isolated vertebral defects was used to test the biomechanical properties of the bone cements.

## 2. Results

**Figure 1:**
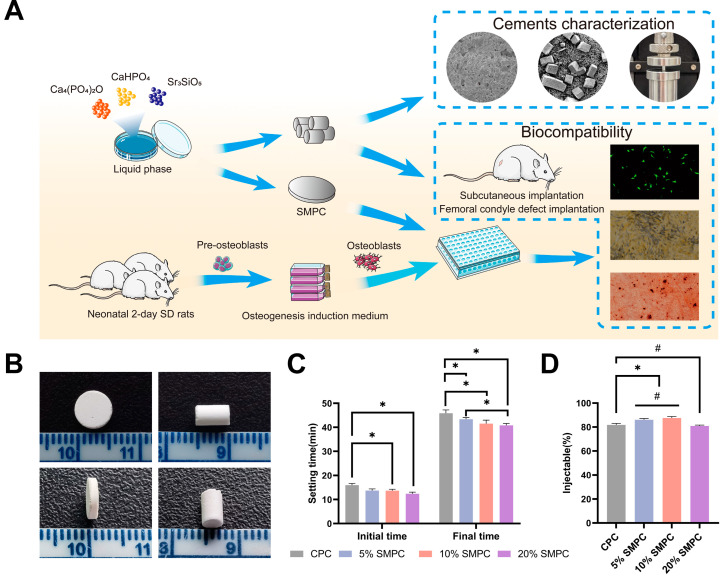
Schematic illustration of characterization and biocompatibility testing of bone cement (**A**). Solid cement samples were prepared for cement characterization testing and cell culture (**B**). Initial and final setting time of CPCs and SMPCs (**C**). Performance of injectability of bone cements (**D**) (*: *p* < 0.05; #: *p* > 0.05).

The study of strontium-modified calcium phosphate bone cement is depicted in a simplified schematic format in Figure 1A. The addition of tristrontium silicate enhanced the bioactivity of the calcium phosphate bone cement, while the extent of inflammation caused by the bone cement was observed by subcutaneous implantation. Finally, SMPCs were found to enhance the biomechanical properties of bone, as shown by mechanical testing of bone cements implanted in fresh isolated bones.

### 2.1. Cement Production, Setting Time, and Injectability

Cement formulations of different proportions were harmonized into a paste at a liquid-to-powder ratio of 0.45 mL/g and injected into the molds smoothly. The samples were removed from the molds easily and remained intact (Figure 1B). The 20% SMPC cements took slightly longer to blend, while the other groups did not show different properties. The initial and final setting times of the cement paste in the SMPC and CPC groups were examined separately (Figure 1C). The setting time of SMPC decreased simultaneously with the increase in the addition of tristrontium silicate. For the initial setting time, the setting time of CPC was not significantly different from that of 5% SMPC, while the setting time of SMPC with 10% and 20% tristrontium silicate addition was statistically significant compared to CPC. The reduction in setting time due to tristrontium silicate addition was amplified in the final setting time: all three groups of SMPCs had statistically significant last setting times compared to CPC. The final setting time of CPC was (45.9 ± 1.4) min, while the final setting time of 20% SMPC decreased to (40.8 ± 0.8) min. The performance of several bone cements in terms of injectability is shown in Figure 1D. We found that adding small doses of tristrontium silicate enhanced the injectability of bone cements. The injectability of both 5% SMPC and 10% SMPC showed an increase compared to CPC (*p* < 0.05). Meanwhile, there was no significant difference between 5% SMPC and 10% SMPC injectability compared to CPC. However, the injectability of the cement showed a decrease compared to CPC when the addition percentage of strontium trisilicate was 20% (*p* < 0.05). The injectability performance of cements declines when the quantity of tristrontium silicate additive approaches 20%; the injectability is not different from that of CPC, but the difference is statistically significant compared to 10% and 5% SMPCs.

### 2.2. Phase Composition

**Figure 2:**
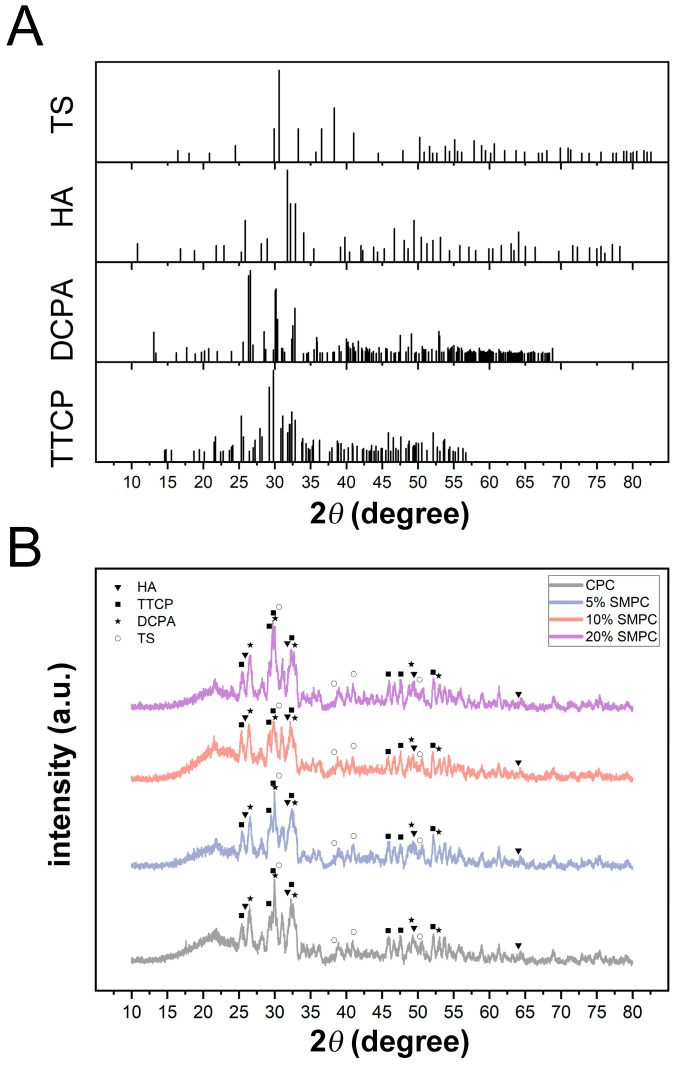
Theoretical peak positions of Sr_3_SiO_5_ (tristrontium silicate, PDF#26-0984), tetracalcium phosphate (Ca_4_(PO_4_)_2_O, PDF#25-1137), dicalcium phosphate anhydrous (CaHPO_4_, PDF#77-0128), and hydroxyapatite (Ca_5_(PO_4_)_3_OH, PDF#09-0432) (**A**). XRD diffraction pattern of CPCs and SMPCs after being ground into a homogeneous powder (**B**).

Calcium phosphate cements typically consist of a solid phase comprising one or more calcium phosphate compounds and a liquid phase providing a fluid environment for the solid phase reaction. TTCP and DCPA are typical solid-phase components in CPC, and it was proven that they produced hydroxyapatite (HA) through the reaction of Equation (1) [12]. Samples were crushed into a fine powder and then subjected to XRD to analyze the phase composition of the CPC and SMPC following the hydration reaction. Comparing the theoretical diffraction peaks of Figure 2A with those predicted theoretically revealed that HA was generated in the initial stage in the hydrated products, with the TTCP, DCPA, and TS also present in the final products in all groups. The addition of TS did not significantly affect the phases in the diffraction patterns, despite the dominant peak of the SMPC diffraction patterns shifting. Since strontium has a larger ionic radius than calcium ions, the addition of tristrontium silicate to calcium phosphate bone cement can cause the calcium portion of the hydroxyapatite end product to be replaced by strontium, leading to changes in the crystal structure of hydroxyapatite.

(1) $$2{\mathrm{Ca}}_{4}{\left({\mathrm{PO}}_{4}\right)}_{2}O+2{\mathrm{CaHPO}}_{4}\to {\mathrm{Ca}}_{10}{\left({\mathrm{PO}}_{4}\right)}_{6}{\left(\mathrm{OH}\right)}_{2}$$

### 2.3. Microstructure and Mechanical Properties of Cements

**Figure 3:**
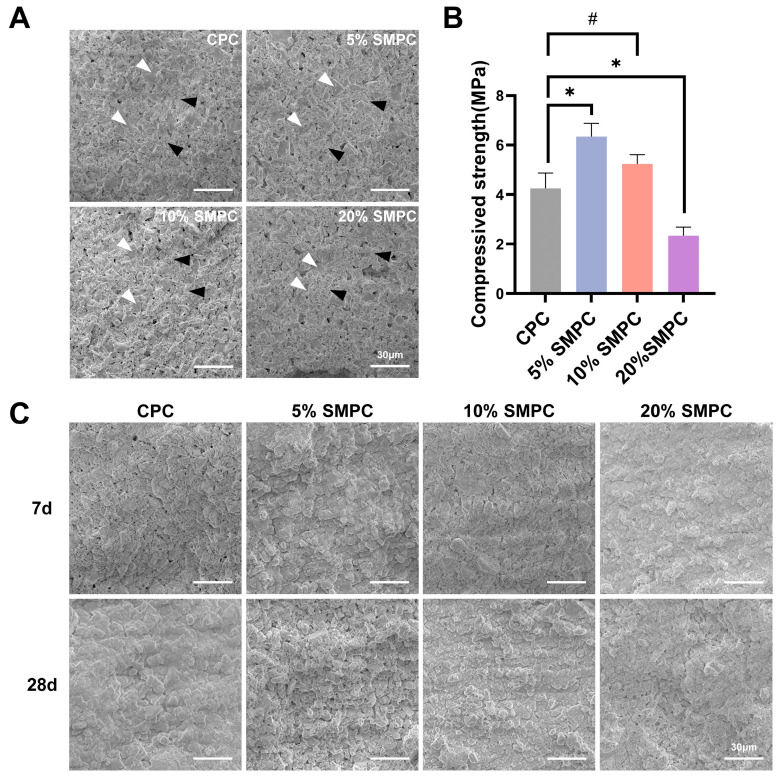
The SEM micrographs of CPCs and SMPCs ((**A**) White arrow: large lamellar structure; black arrow: minute patchy structure). Measurement of the compression strength of CPCs and SMPCs (**B**). SEM micrographs of CPCs and SMPCs immersed in SBF for 7 and 28 days (**C**) (scale bar = 30 μm; *: *p* < 0.05; #: *p* > 0.05).

The SEM images of the cement samples showed varying degrees of lamellar structure formation on their surfaces, and microporous-like structures could be seen (Figure 3A). The main hydration products of calcium phosphate bone cements are minute patchy structures and some incompletely hydrated large lamellar structures. In addition, the results of compression strength testing of CPC and SMPC cylindrical cement samples were shown in Figure 3B. The addition of 5% tristrontium silicate significantly enhanced the mechanical properties of the bone cement compared to CPC alone. However, increasing the percentage of tristrontium silicate to 10% or 20% showed a decrease in the mechanical properties of the cement. The surface morphology of each group of cements soaked in SBF species for 7 and 28 days is shown in Figure 3C. The pictures showed that the crystalline precipitation on the surface of both cements increased with the prolongation of mineralization time, which was more apparent in SMPC. After mineralization, all cements became less porous on the surface, and the samples were more compact. As the proportion of tristrontium silicate increased, the lamellar crystals on the surface of the material cross-linked into a mesh-like structure, indicating that the content of tristrontium silicate correlates with the change in cement structure. The EDX results show that the crystals precipitated contain mainly four elements: calcium (Ca), phosphorus (P), Sr, and silicon (Si). The Ca/P ratio gradually increased from 1.67 to 1.90 with the addition of tristrontium silicate, which predicts that the crystals may be the precipitates of HA and tristrontium silicate (Table 1).

### 2.4. Degradation and Biomineralization In Vitro

In order to explore the degradation of the cements in vitro, different groups of bone cements were immersed in PBS. The bone cements of each group were immersed in PBS for 35 days and dried, and many granular crystals were found on the surface of SMPC but not on the surface of CPC. In addition, the precipitated particles were more evident in the 5% and 10% tristrontium silicate addition groups, especially in the 10% group. The same crystals precipitated in the 20% SMPC group but were significantly less common. With constant magnification with scanning electron microscopy, prismatic crystal particles of different sizes were clearly seen embedded on the surface of SMPC. Further magnification revealed that in the 20% SMPC group, the precipitated crystal particles were smaller than those in the 5% SMPC group and 10% SMPC group. One possible explanation is that the cement samples’ dissolution–deposition reaction can only occur in an aqueous environment, and the reaction stops when the cement dries. The PBS environment triggered a second round of reaction for the unreacted part of the original powder. It is certain that strontium ions are involved in the dissolution–deposition reaction and show accelerated reaction at 5% and 10% addition rates. The 20% addition rate makes this accelerated trend decrease. However, the specific mechanism of this has not been studied in depth.

**Figure 4:**
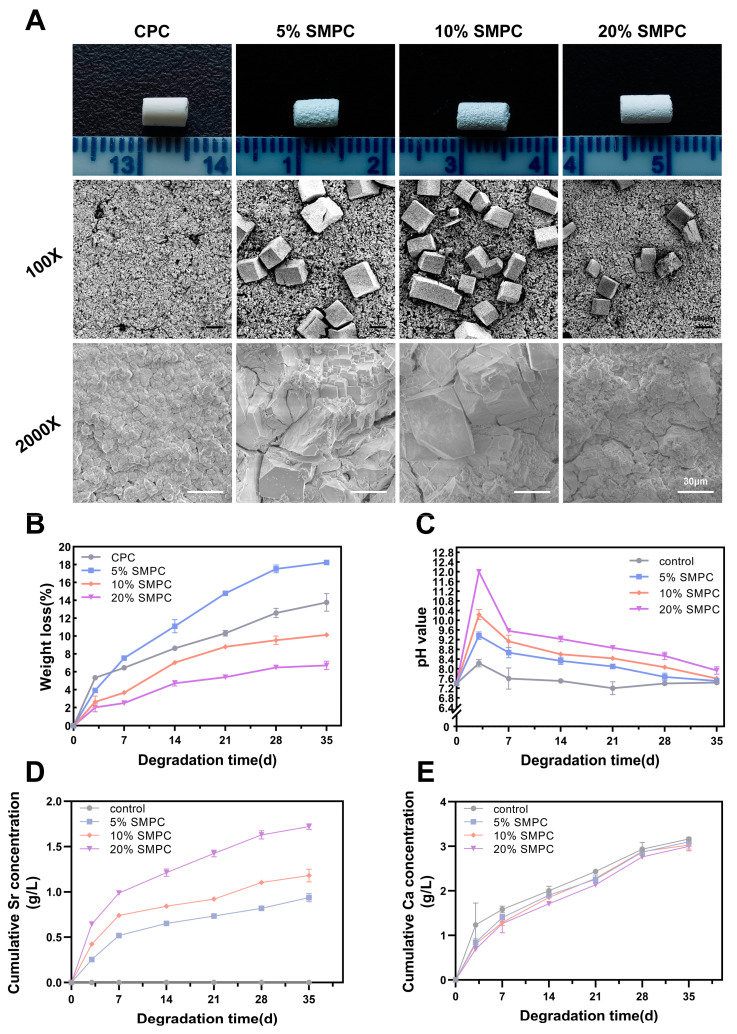
Appearance of CPC and SMPC immersed in PBS solution ((**A**) scale bar = 30 μm). Weight loss of CPC and SMPC immersed in PBS solution (**B**). pH (**C**), cumulative Sr concentration (**D**), and cumulative Ca concentration (**E**) of PBS solution during the soaking of cements.

The weight loss rates of CPC and SMPC samples are presented in Figure 4B. The highest rate of weight loss was observed in the 5% SMPC group (18.24 ± 0.22%) compared to (13.78 ± 0.99%) in the CPC group. In general, the weight loss rate was lower in the three SMPC groups than that in the CPC group, and the degradation rate decreased with increasing doses of tristrontium silicate, except for the 5% SMPC group. Furthermore, the degradation rate of cement in the 5% SMPC group was also lower than that in the CPC group in the first three days; after that, it started to increase gradually and exceeded the CPC group on the seventh day. As the soaking time for CPC increased, the pH of the PBS solution decreased. The pH value of the PBS solution gradually increased in the first 3 days and reached a maximum on the third day; after that, it gradually declined and tended to be neutral. However, the pH of SMPCs was slightly higher than that of CPCs (Figure 4C). Similarly, the strontium in SMPC was released gradually and moderately over 35 days, with the amount of strontium released increasing with the amount of tristrontium silicate added (Figure 4D). Figure 4E shows the gradual release of Ca ions from the bone cement over 35 days of immersion and shows that the release of Ca does not appear to be affected by the addition of tristrontium silicate.

### 2.5. Toxicity and Adhesion Assessments

**Figure 5:**
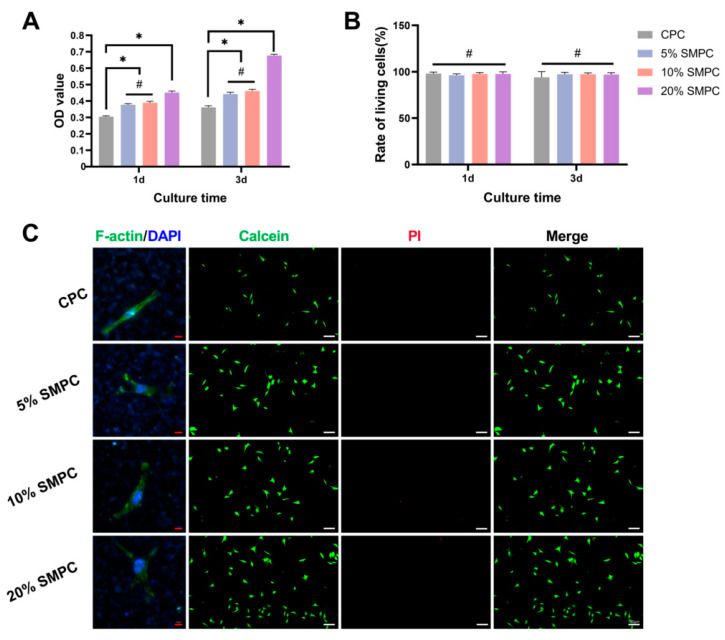
Proliferation of osteoblasts in conditioned medium culture (**A**). Live-dead staining ((**C**) white scale bar = 30 μm) and quantification (**B**) of osteoblasts in culture together with bone cement. Adhesion of osteoblasts inoculated on the surface of bone cement for 1 day ((**C**) red scale bar = 30 μm) (*: *p* < 0.05; #: *p* > 0.05).

The proliferation numbers of osteoblasts in each group of bone cement material for 1 and 3 days of culture are shown in Figure 5A. Cell counts in the CPC were already much lower than those in the SMPCs by day 1, and the impact of boosting osteoblast proliferation grew more obvious as the amount of tristrontium silicate added increased. However, the cell numbers had no significant difference between the 5% SMPC and 10% SMPC groups. This may be because the 5% and 10% tristrontium silicate dose changes were not significant. A substantial increase in the number of cells was observed in the 20% SMPC group. Whether tristrontium silicate was toxic to osteoblasts was analyzed with live-dead staining and quantitative analysis (Figure 5B,C). There was no significant difference in the viable cell rate between the groups, indicating that tristrontium silicate at different ratios did not cause toxicity to osteoblasts. Osteoblasts were seeded directly on the cement surface and stained with phalloidin after 1 day. As the strontium trisilicate content increased, osteoblasts spread out and attached more firmly to the bone cement (Figure 5C). These results suggest that strontium silicate enhances the adhesion of osteoblasts to bone cement.

### 2.6. Proliferation and Osteogenic Capability Test

**Figure 6:**
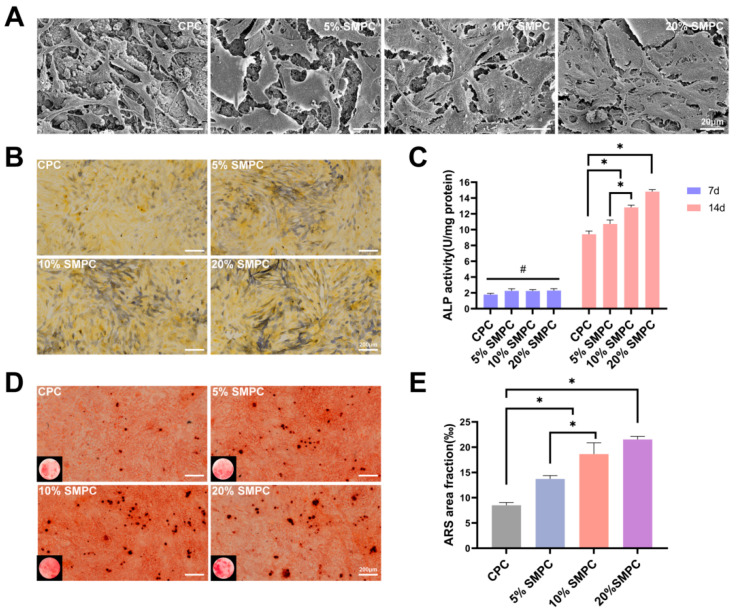
SEM images of osteoblasts cultured on the surface of bone cement for 3 days ((**A**) scale bar = 20 μm). ALP staining ((**B**) scale bar = 200 μm) and quantification (**C**) of osteoblasts cultured together with bone cement for 14 days and ARS staining ((**D**) scale bar = 200 μm) and quantification (**E**) at 28 days (*: *p* < 0.05; #: *p* > 0.05).

**Figure 7:**
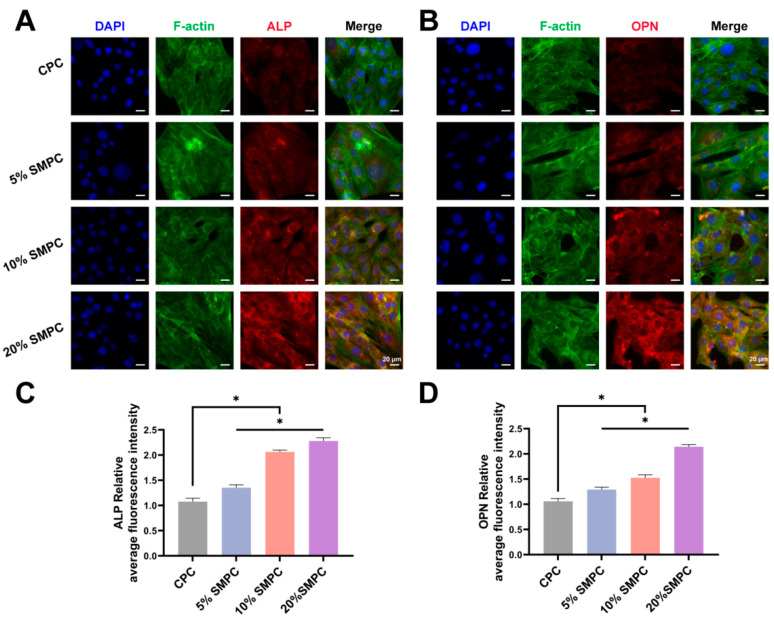
ALP ((**A**) scale bar = 20 μm) and OPN ((**B**) scale bar = 20 μm) fluorescence staining images of osteoblasts cultured together with bone cement for 14 days. Quantification of average fluorescence intensity of ALP (**C**) and OPN (**D**) fluorescent stained images (*: *p* < 0.05).

Images of cultivated osteoblasts on the cement sample surface after 3 days revealed much-improved cell adhesion to the bone cement. Compared with the CPC group, osteoblasts in the SMPC group had a larger extension area, and a large number of cellular pseudopods could be observed (Figure 6A). The ALP activity was influenced by both cellular osteogenic activity and total number of cells, and the results of the ALP staining and assay are shown in Figure 6B,C. The ALP activity of CPCs and SMPCs gradually increased with increasing co-culture time. ALP is a marker of early osteoblast maturation, and tristrontium silicate enables SMPCs to stimulate osteoblast ossification. Mature osteoblasts secrete calcium ions outside the cell to form calcium salt deposits, a critical bone formation process. Alizarin Red S staining displayed calcium nodules that had been deposited extracellularly. As seen in Figure 6D, SMPCs aided in the osteoblasts’ ability to release calcium ions. More importantly, this promotion effect increases simultaneously with the addition of tristrontium silicate. ALP and OPN are important bio-markers of the transition of osteoblasts from a naive to a mature state. After the bone cement samples and osteoblasts were cultured together for 14 days, we performed immunofluorescence staining of osteoblasts for ALP and OPN (Figure 7A,B). The amount of ALP and OPN in osteoblasts increased with the proportion of tristrontium silicate in the bone cement. The mean fluorescence intensities of the different groups were statistically different (*p* < 0.05). The highest mean fluorescence intensities of ALP and OPN were found in 20% SMPC.

### 2.7. Local Inflammatory Response

**Figure 8:**
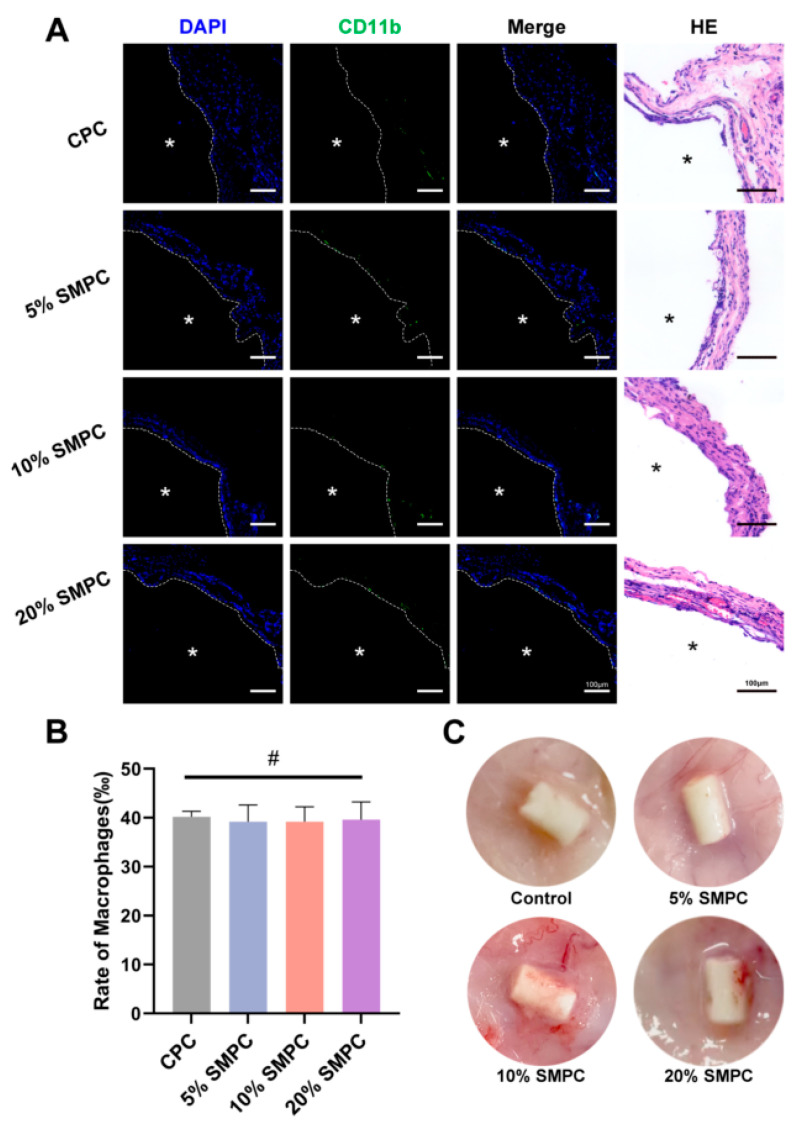
H&E staining and immunofluorescence staining of subcutaneous implantation of bone cement in SD rats for 14 days ((**A**) scale bar = 100 μm; *: side of the bone cement). Macrophage ratio in immunofluorescence staining (**B**). Rat subcutaneous implantation model (**C**) (#: *p* > 0.05).

Preformed cement columns were implanted subcutaneously in SD rats to assess whether different cement groups caused local inflammatory responses (Figure 8C). Both the CPC and SMPC groups experienced no local edema or poor wound healing after 14 days. H&E staining showed hyperplasia of fibrotic tissue around the cement block but no obvious macrophage infiltration (Figure 8A). Immunofluorescence staining further clarified the amount of migrating macrophages in the tissue (Figure 8A). Quantitative analysis of macrophage proportions revealed no significant differences in the CPC and SMPC groups (Figure 8B). This implies that the addition of tristrontium silicate did not increase the pro-inflammatory properties of the bone cement.

### 2.8. Osteogenic Ability Assessment In Vivo

**Figure 9:**
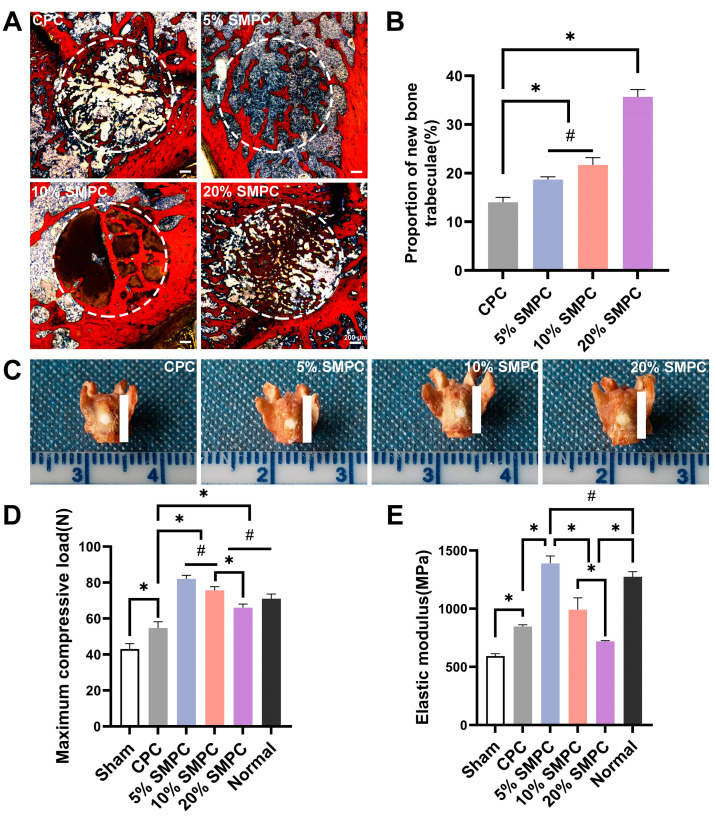
VG staining images ((**A**) scale bar = 20 μm) and quantitative analysis (**B**) of femoral condylar defects (the area shown by the white dashed line is the femoral condylar defect). Fresh isolated bone defect model (**C**); scale bar = 6 mm). Maximum compressive load (**D**) and elastic modulus (**E**) of isolated bones (*: *p* < 0.05; #: *p* > 0.05).

Femoral condylar defects in SD rats were used to investigate the osteogenic effect of bone cement in vivo. The new bone trabeculae grew into the void created by the degradation of bone cements. VG staining of the femoral condyles showed that the number of new bone trabeculae was higher in the group with strontium-silicate-modified calcium phosphate bone cement than in the CPC (Figure 9A,B). There was no significant difference between 5% SMPC and 10% SMPC, but the 20% SMPC was significantly higher compared to both (*p* < 0.05).

### 2.9. Biomechanical Properties of Isolated Bone

Considering that bone cement is implanted locally for bone defects, we performed bone cement injection into the fresh isolated bone to investigate whether local implantation of bone cement would affect the bone’s mechanical properties (Figure 9C). The maximum load reflects the structural mechanical properties of the bone and modulus of elasticity as an assessment of the properties of bone materials. The maximum compressive force and elastic modulus of the isolated bone for each group are shown in Figure 9D,E. Filling with the cement significantly elevated the maximum compressive load of the elastic modulus of the defective bone. Furthermore, the addition of strontium silicate facilitated the increase in the maximum compressive load and the elastic modulus of the bone. However, as the amount of strontium silicate increased, this elevation effect decreased. The appropriate addition of tristrontium silicate can contribute to the improvement of both the material and structural mechanics of bone.

## 3. Discussion

In this study, we successfully incorporated bioactive tristrontium silicate components into CPCs to create a unique strontium-modified phosphate cement with varying strontium concentrations. SMPC has adequate mechanical properties, reaching a compressive strength of 6.00 ± 0.74 Mpa as the proportion of tristrontium silicate increases to 5%. Additionally, SMPC is capable of generating a sustained release of active Sr ions, with minimal influence on the surrounding pH. SMPCs significantly promote the proliferation and maturation of osteoblasts by culturing together with osteoblasts in vitro. In vivo subcutaneous implantation of cements in SD rats showed that adding tristrontium silicate to the SMPC groups did not result in a significant local inflammatory response compared to CPC. SMPCs also exhibited enhanced bone regeneration in femoral condylar defect implantation. Because of their excellent mechanical properties and bioactivity, SMPCs have great potential for use in tissue engineering.

Tristrontium silicate, a compound with high strontium content, did not show significant differences compared with CPC during cement blending. The paste-like SMPC precursors can be injected into the molds smoothly and demolded intact after solidification. The setting time of SMPC tends to be prolonged with the increase in strontium ratio. This may be because the particles of tristrontium silicate retard the dissolution–recrystallization process of TTCP and DCPA. Lode et al. found a similar prolongation of the setting time by adding SrCO_3_ to the β-TCP cement reaction system [34]. Proper setting time is necessary for the application of bone cement, and factors such as particle size, liquid to powder ratio, ambient temperature, etc., can affect the setting time [41]. The optimal setting time of SMPC can be achieved by altering the amount of tristrontium silicate added to make it more operator-friendly. The mechanical strength of the bone cement is an important indicator, and good bone cement strength should be close to that of cancellous bone, within approximately 30 MPa [42,43]. However, patients with osteoporosis are unable to withstand slightly higher compressive stresses due to the decreased bone mass of cancellous bone [44]. This means that an implant with the appropriate compressive load for a normal person may cause a local re-fracture for such patients. The compressive strength of SMPCs showed a trend of small doses of tristrontium silicate addition enhancing the compressive strength; this boosting effect started to decay with increasing the amount of additives. The compression strength was much lower than that of CPC even after adding 20% tristrontium silicate. We expect that the tristrontium silicate present in low concentrations (<20%) will be more widely dispersed throughout the final system of the bone cement hydration process. In comparison, tristrontium silicate added at higher concentrations (≥20%) will be more widely dispersed, which may disrupt the relatively stable product system of the hydration reaction, leading to a reduction in mechanical characteristics. A bone cement that is too weak mechanically will not support compressive stresses under physiological conditions, while one that is too strong will increase the risk of fracture in adjacent bone tissue. Therefore, 5% SMPC is a more appropriate ratio, with a compression strength of 6.00 ± 0.74 MPa. The mechanical strength of 5% SMPC was increased by 20% compared to the CPC group. However, there is some debate in the literature on whether or not the Sr ion substantially impacts the mechanical characteristics of bone cement after being incorporated. Several studies have shown that the introduction of strontium promotes the mechanical properties of bone cement, with different proportions of strontium-containing compounds exhibiting other promotive effects [34,45,46]. In contrast to our findings, Wu et al. reported that adding SrCO_3_ to bone cement had no appreciable effect on the cement’s compressive strength [47]. Multiple studies have confirmed this phenomenon [48,49]. Many factors affect bone cement’s mechanical properties, including the solid–liquid ratio, porosity, cement composition, etc. [41]. Tristrontium silicate contributes to the compressive strength of bone cement because of its unique chemical properties. This strength comes from the whole compound, not just the Sr ion, which may be a reasonable explanation.

For the application of calcium phosphate bone cement to be realized, the problem of bioactivity must be addressed. We improved bone cement’s bioactivity and ensured superior osteoinductive properties by incorporating bioactive strontium into the solid phase. Such a facilitation effect was already present in the 5% addition of tristrontium silicate and was further enhanced in the 10% and 20%. Many previous studies on the osteogenesis-promoting effect of strontium have been verified with bone marrow stem cells [34,47]. We selected osteoblasts to study the effect of bone cement on their osteogenic effect. We observed that tristrontium silicate significantly stimulated cell proliferation and promoted osteogenesis dose-dependently when osteoblasts were seeded onto the bone cement. In addition to strontium, the biological roles of many reactive metal ions have been determined. Both copper and zinc have been shown to promote bone growth and repair [31,50]. Since strontium and calcium are in the same group in the periodic table, they all belong to the alkaline earth metal group. It has been found that the effect of strontium on osteoblasts may be through the activation of calcium-sensitive receptors (CaSR), which means that strontium has an inherent advantage over other metal ions when it comes to its biological role [51,52]. Similar results were reported by Wu et al., who concluded that strontium had no appreciable effect on the proliferation of MC3T3-E1 but dramatically boosted the mobility and cell spreading area of HUVECs [47]. In fact, many factors influence cell proliferation. There may be differences in the effects of bone cement extracts and bone cements on cells, with strontium ions being continuously released by bone cements incorporated with strontium. Whether strontium has a role in promoting cell proliferation is controversial and may be related to the overall composition of strontium-containing bone cements. Not only are strontium cations present in the culture system provided by the extracts, but different anions are present. For the osteoblast proliferation-promoting effect exhibited by tristrontium silicate, we attribute this to the silicate anion. Considering the practical application of bone cement, we verified whether SMPC triggers inflammation at the implant site. After 14 days of subcutaneous implantation, there was no statistically significant difference between SMPC and CPC in the local macrophage ratio. This has been overlooked in many studies, bearing in mind that implants that cause a violent inflammatory storm can be catastrophic for local bone regeneration and reconstruction [53]. SMPCs also exhibited superior bone regeneration compared to CPCs in the femoral condylar defect implantation model. However, the difference between the 5% SMPC and 10% SMPC new bone trabeculae was not significant, which may be due to the fact that the change in the percentage of tristrontium silicate addition was not significant.

The results of this study indicated that the proposed method of incorporating tristrontium silicate into calcium phosphate bone cement is workable and that the addition rate of 5% yielded a considerable improvement in mechanical and biological properties. It also had the most rapid passive degradation rate, reaching 18.2% by day 35. We also confirmed the osteogenic effect of SMPC with in vitro experiments. Our research provides a new formulation for the application of bone cement. By changing the composition of the solid phase through introducing tristrontium silicate, a simple solid–liquid mixture is all that is required to achieve the formation of SMPC that promotes bone growth in the process of filling bone defects. SMPC has much potential for future clinical applications owing to its good injectability, mechanical load support potential, and osteogenesis properties.

However, our study had some limitations. We did not investigate how SMPC works at the molecular level to promote osteogenesis; this will be addressed in the future

## 4. Materials and Methods

### 4.1. Fabrication of the CPC and SMPC

In this experiment, we used tetracalcium phosphate (Ca_4_(PO_4_)_2_O, TTCP), dicalcium phosphate anhydrous (CaHPO_4_, DCPA), and tristrontium silicate (Sr_3_SiO_5_, TS) powders to prepare the strontium-modified phosphate cement (SMPC). Tristrontium silicate powder was generated with strontia (SrO) and distrontium silicate (Sr_2_SiO_4_) through a solid-phase reaction at 1475 °C. All the powders were analytical grade and bought from Suzhou dinganTec Co., Ltd. (Suzhou, China).

TTCP, DCPA, and TS were mixed at different ratios (Table 2), according to previous studies in our laboratory [54]. CPC contained 72.91%(wt) TTCP and 27.09%(wt) DCPA. TS was incorporated into CPC with a weight of 5.00%(wt), 10.00%(wt), and 20.00%(wt), respectively. The liquid phase included 0.1 mol/L NaH_2_PO-Na_2_HPO, 0.8%(wt) sodium alginate, and 0.5 mol/L citric acid. The final pH of the solutions was adjusted to 7.0 with sodium hydroxide. The liquid-to-powder ratio (l/p) was 0.47 mL/g. The cement paste was injected into the polytetrafluoroethylene molds to prepare the cylinder samples (Ø3 × 5 mm for cement characterization) and disc samples (Ø6 × 1 mm for cell culture experiments). The cement paste was demolded after 1 h of solidification in the polytetrafluoroethylene mold and dried at room temperature for 24 h for subsequent experiments.

### 4.2. Characterization of Cement

#### 4.2.1. Setting Time and Injectability

The CPC and SMPC pastes were injected into a tetrafluoroethylene mold. Samples were tested at different times using a Vicat apparatus consisting of a frame with a rod weighing 300 g and a 1 mm stainless steel needle at the end. When the initial setting test needle sank to 5 mm from the bottom plate, this was determined as the initial setting time of the cements. The time until the needle was unable to penetrate more than 1 mm into the sample was used to determine the final setting time. Each experiment was performed in triplicate, and the average value was calculated.

The injectability of the cement pastes was assessed in vitro as follows. The cement pastes were prepared as described above by blending for 1 min (starting with adding the curing solution). Then, the contents were transferred into a 1 mL syringe with a 1.7 mm needle. We used mechanical testing equipment to extrude the cement paste at a rate of 5 mm/min with a force of 150 N until the cement paste could not be extruded. The following equation was used to calculate the percentage of injectability of the cement:

$$\mathrm{Injectability}\left(\%\right)=\frac{W_{0}-W_{1}}{W_{0}}$$

where *W*_0_ is the initial mass of cement in the syringe (g); *W*_1_ is the mass of cement remaining in the syringe after extrusion (g).

Each group of assays was repeated 6 times, and the mean of each group was calculated for comparison.

#### 4.2.2. X-ray Diffraction and Mechanical Characterization

Cement samples (ground to powder) dried at room temperature for 24 h were used to measure the X-ray diffraction (XRD) patterns using a SHIMADZU XRD-6100 from Shimadzu Corporation (Kyoto, Japan) according to ISO 13175-3 standard. Data were gathered in the range 2θ = 10°–80° with a step size of 0.02° and a normalized count time of 1.5 s per step. The XRD patterns were analyzed using MID jade 6.0. The International Centre for Diffraction Data’s Joint Committee on Powder Diffraction Standards (JCPDS) reference patterns were used to verify phase composition. The compressive strength of cylinder samples was analyzed with a load rate of 1 mm/min using a universal testing machine (Model 2519-105, INATRON; Norwood, MA, USA) according to the ISO 13175-3 standard. During the measurement, we recorded the movements of compression plates to estimate strain. The following equation was used to determine the compressive strength:

$$\sigma =\frac{4P}{\Pi D^{2}}$$

where σ is the compressive strength (MPa); *P* is the compressive load (N); *D* is the sample diameter (mm).

Each group of assays was repeated 6 times, and the mean of each group was calculated for comparison.

#### 4.2.3. In Vitro Degradation and Biomineralization

The CPC and SMPC samples were immersed in PBS (Phosphate-Buffered Saline) solution (pH 7.4) at 37 °C with a solid/liquid mass ratio of 0.1 g/mL. The PBS was changed once a week. The PBS solution was collected to analyze the ionic release of the cements using inductively coupled plasma optical emission spectrometry (ICP-OES; Aglient-5110, Agilent; New York, NY, USA) and the pH change of the solution according to the ISO 13175-3 standard. At different time points, the specimens were removed from the liquid, rinsed with distilled water, and dried in the oven at 50 °C for 4 h. The percentage of weight loss was calculated as follows:

$$\mathrm{Weight}\;\mathrm{loss}\left(\%\right)=\frac{W_{0}-W_{t}}{W_{0}}$$

where *W*_0_ is the starting dry weight; *W_t_* is the dry weight at time t.

A modified simulated body fluid (m-SBF; Shanghai yuanye Bio-Technology Co., Ltd., Shanghai, China) was prepared and used as a biomineralization medium. Samples of CPC and SMPC were submerged in m-SBF at a concentration of 4 g/L for 28 days, with fluid changes every 7 days. Biomineralization was assessed using scanning electron microscopy (SEM; S-3400N, Hitachi; Hitachi, Japan). Samples were gently washed three times in deionized water and finally observed. Elemental composition was analyzed using Energy Dispersive X-Ray Analysis (EDX; JEOL 7800F, JEOL Ltd.; Tokyo, Japan). At least 3 samples from each group were randomly selected for testing.

### 4.3. Biocompatibility and Osteogenic Differentiation In Vitro

#### 4.3.1. Osteoblast Culture

Fragments of cranial bone were obtained from 2-day-old SD rats, washed in sterile PBS, and incubated at 37 °C in a 5% CO_2_ environment with 0.25% Trypsin for 10 min. Then, they were neutralized with a culture medium, and the collagenase I digestion continued for another 90 min while being stirred and shaken every 20 min. A medium was added to neutralize the reaction at the end of the digestion.

Next, the cell suspension was passed through a 20 μM strainer to obtain a uniform single-cell suspension. The cells were resuspended in osteogenesis induction medium, including Dulbecco’s modified Eagle medium (DMEM; Gibco, Thermo Fisher Scientific; Waltham, MA, USA) supplemented with 10% heat-inactivated fetal bovine serum (FBS; Gibco, Thermo Fisher Scientific; Waltham, MA, USA), 10 mmol/L β-Glycerol phosphate disodium, 0.1 umol/L dexamethasone, 50 μg/mL phosphoascorbate, 100 U/mL penicillin, and 50 μg/mL streptomycin. Cells were counted using a Beckman Coulter Z1 Particle Counter (Beckman Coulter; Fullerton, CA, USA) and then cultured in 100 mm plates, grown to confluence, and harvested for subsequent experiments.

Conditioned medium: All cement samples were soaked overnight in 75% alcohol, washed 3 times with DMEM, and dried naturally under UV light. Then, bone cements were soaked in DMEM at 0.2 g/mL for 24 h to obtain the extract according to the ISO 13175-3 standard. Subsequently, other ingredients were added according to the ratio of the aforementioned osteogenic medium, and the osteogenic conditioned medium was formulated for the corresponding cements.

#### 4.3.2. Cell Proliferation and Viability

Cells were seeded onto 96-well plates at a density of 0.2 × 10^4^ cells per well and incubated at 37 °C and 100% humidity with 5% CO_2_. After 24 h, all cells were grown adherently, and the medium was removed, washed 3 times with PBS, and added to the conditioned medium for 1 or 3 days. The cell proliferation was evaluated with a Cell Counting Kit-8 (CCK-8; c0038, Beyotime; Shanghai, China). The cells were placed in a culture medium containing CCK-8 and incubated in a humidified atmosphere at 37 °C for 1 h. The optical density (OD) of the chromophore was measured at 450 nm using an enzyme-linked immunoadsorbent assay plate reader (Thermo Fisher Scientific; Waltham, MA, USA).

A Calcein/PI Cell Viability/Cytotoxicity Assay Kit (C2015S, Beyotime; Shanghai, China) was used to evaluate the cytotoxicity of SMPC for cells. Cells were inoculated on 24-well plates with cement discs at a density of 0.5 × 10^4^ cells per well. Then, the cells were cultured at 37 °C and 100% humidity with 5% CO_2_ for 1 or 3 days. After that, the cells were incubated for 30 min with Calcein AM/PI Detecting Working Solution. The images were then captured using a fluorescent microscope (Leica; Weztlar, Germany). The quantity of live/dead cells was counted with Image J software (Image J 1.53r, National Institutes of Health; Bethesda, MD, USA). A total of six randomly selected pictures were analyzed to determine the percentage of viable cells.

#### 4.3.3. Alkaline Phosphatase (ALP) Activity and Staining

The alkaline phosphatase (ALP) activity was determined using an Alkaline Phosphatase Activity Assay (Nanjing Jiancheng Bioengineering Institute). The p-nitrophenol solution was used as the standard, and the standard curve was plotted. On days 7 and 14 when cells and samples were cultured together, lysate was added to each well, and the supernatant was taken after centrifugation. Then the assay buffer was added and mixed gently by blowing, and the reaction was terminated by adding the reaction termination solution after incubation at 37 °C for 10 min. The absorbance was measured at 405 nm.

For ALP staining, cells were cultured together with disc cements as described in Section 4.3.2. for 14 days. After removal of the medium and washing with PBS, cells were fixed with 4% paraformaldehyde for 15 min. Cells were stained with ALP Incubation Solution (Solarbio; Beijing, China) for 30 min after being rinsed three times with PBS. The ALP-stained images were obtained using bright field microscopy (Leica; Weztlar, Germany).

#### 4.3.4. Cell Adhesion and Microstructure

Cells were cultured on the material’s surface for 1 day to detect the adhesion of osteoblasts. The cells were fixed with 4% paraformaldehyde solution for 15 min, then permeabilized with 0.5% Triton X-100 solution for 5 min. The 500 μL phalloidin (Solarbio; Beijing, China) at a concentration of 100 nM was used to cover the cells on the material’s surface for 30 min at room temperature in the dark. After washing with PBS buffer for 10 min, 500 μL of DAPI solution (100 nM) was used to re-stain the nuclei for 15 min. The material was washed with PBS, and the rhodamine excitation filter and DAPI excitation filter were selected for observation under a fluorescent microscope (Leica; Weztlar, Germany) for observation and photography. Similarly, cells were cultured on the material’s surface for 3 days. The cells were rinsed twice with PBS buffer and fixed in a 4% paraformaldehyde solution at room temperature for 15 min. The samples were then subjected to cascade dehydration. The surface of the samples containing the cells was treated with gold spray and photographed using an SEM (S-3400N, Hitachi; Hitachi, Japan).

#### 4.3.5. Alizarin Red S Staining

After 21 days of culturing the cells with bone cement material, the cells were gently washed with PBS buffer and then fixed in 4% paraformaldehyde. The cells were stained with Alizarin Red S dye for 30 min at room temperature. After washing with PBS, the stained cells were observed under the microscope (Leica; Weztlar, Germany).

#### 4.3.6. Immunofluorescence Analysis

Cells were cultured together with bone cement material for 14 days, the medium was removed, and cells were washed lightly with PBS 3 times. Cells were fixed with 4% paraformaldehyde for 10 min and then soaked with 0.1% Triton X-100 for 15 min to make the cell membrane permeable. Subsequently, cells were incubated with primary mouse anti-alkaline phosphatase (ALP, 1:500, Abcam, UK) or mouse anti-osteopontin (OPN, 1:500, Abcam; Cambridge, UK) for 12 h at 4 °C after blocking with bovine serum albumin (BSA) for 1 h. After washing 3 times for 5 min each in PBS, Alexa Fluor 594-labeled donkey anti-mouse (1:1000, Abcam; Cambridge, UK) was incubated for 2 h at 37 °C in the dark. Cells were then washed with PBS and incubated with DAPI (40,6-diamidino-2-phenylindole, Beyotime; Shanghai, China) solution and phalloidin (Solarbio; Beijing, China) for 30 min. After washing with PBS, the stained cells were observed under the microscope (Leica; Weztlar, Germany).

### 4.4. In Vivo Experiments

All animal experiments were conducted in accordance with Xi’an JiaoTong University guidelines for animal research and use.

#### 4.4.1. Animal Surgery

Subcutaneous implantation: Sprague Dawley (SD) rats (*n* = 24, 2-month-old female rats; 6 per group) were anesthetized with an injection of pentobarbital (Nembutal, 30 mg kg^−1^) intraperitoneally. A sagittal incision of approximately 1 cm was created on the back of the rats, and then the subcutaneous layer was fully separated. Different cylindrical cement samples (Ø3 × 5 mm) were implanted into the loose connective tissue under the dermis. The incisions were tightly sutured.

Femoral condyle defect implantation: An incision of approximately 1.0 cm longitudinally was made through the lateral femoral joint of anesthetized SD rats (*n* = 24, 2-month-old female rats; 6 per group). The femoral condyle was exposed, and a hole 2 mm wide and 5 mm deep was drilled and flushed twice with sterile saline. Different bone cements were blended well and filled into the condylar defect, and the subcutaneous tissue and skin were sutured sequentially. Before implantation, cements used for in vivo experiments were sterilized with gamma radiation.

#### 4.4.2. Biomechanical Testing of Isolated Bone

The SD rats’ lumbar vertebrae (L4) were removed, and a 1.5 mm hole was drilled through the center. Normal L4 vertebrae were used as positive controls. The cavity was filled with different groups of bone cement, and a vertebral compression test was conducted. The intervertebral discs and soft tissues surrounding the vertebral body were removed before testing, leaving only the bone tissue. The upper and lower parts of the vertebral body were polished and made into a standard piece with a height of about 5 mm; it was ensured that the upper and lower parts of the vertebral body were perpendicular to the longitudinal axis of the vertebral body. The compression test was performed using a universal testing machine (Model 2519-105, INATRON; Norwood, MA, USA). The maximum compressive load and elastic modulus were recorded. Each test was repeated 6 times.

#### 4.4.3. Histological and Immunofluorescence Analysis

The subcutaneous tissue of rats was collected 14 days after surgery. For histological analysis, 10% formalin-fixed samples were dehydrated in steps at room temperature before being embedded in O.C.T. (Optimal cutting temperature compound) and cut into 5 μm slices. The sections were then stained using H&E staining according to the manufacturer’s protocol. Stained sections were visualized using a bright-field microscope (Leica; Weztlar, Germany). Immunofluorescence analysis was in accordance with our previously published procedure [55]. Briefly, frozen sections of each sample were stained overnight at 4 °C using a rabbit anti-rat CD11b (1:500, Abcam; Cambridge, USA) antibody as the primary antibody. Then, samples were incubated with Alexa 488-conjugated goat anti-rabbit secondary antibody for 2 h at room temperature in the dark. The secondary staining of cell nuclei was performed using DAPI (40,6-diamidino-2-phenylindole, Beyotime; Shanghai, China). Images were photographed with a fluorescent microscope (Leica; Weztlar, Germany). Six fields of view were randomly selected, and the percentage of total cells occupied by macrophages was calculated using Image J (Image J 1.53r, National Institutes of Health; Bethesda, MD, USA).

After 12 weeks, rats with bone cement implanted in the femoral condyles were euthanized with an overdose of pentobarbital, and the femoral condyles were harvested for evaluation. The femoral condyles were fixed in 4% paraformaldehyde for 2 days and then dehydrated with graded alcohol (70% to 100%). Then, the femoral condyles were embedded in methyl methacrylate, and 30 μm serial sectioning was performed using a Leica 1600 hard tissue sectioning machine. Sections were stained with Van Gieson and examined with a bright-field microscope (Leica; Weztlar, Germany). The proportion of new bone trabeculae (red) occupying the area of the bone defect (white) was calculated using Image pro plus 6.0 (Media Cybernetics, Inc.; Rockville, MD, USA).

### 4.5. Statistical Analysis

Data were collected and analyzed using SPSS Statistics 25.0 (International Business Machines Corporation; Armonk, NY, USA). All values are expressed as mean ± standard deviation. Statistical analysis was performed with one-way analysis of variance (ANOVA), and the Bonferroni method was used for multiple comparisons between groups. A value of *p* < 0.05 was considered statistical significance.

## 5. Conclusions

This study indicates that adding 5% tristrontium silicate to TTCP-DCPA bone cement can produce a new strontium-modified calcium phosphate bone cement with adequate mechanical properties, suitable setting time, and ideal degradability for clinical applications. In addition, in vitro experiments have shown that SMPC has excellent biological effects, and by culturing it together with osteoblasts, SMPC can promote cell proliferation and adhesion and can contribute to the biological effects on the cells. Furthermore, compared to other bone cements, SMPC exhibits better osteogenic properties. Thus, strontium-modified calcium phosphate bone cement offers a new formulation with great potential for clinical applications.

## Figures and Tables

**Table 1:** Elemental composition of CPC and SMPCs.

Sample	Sr (Atomic %)	P (Atomic %)	Ca (Atomic %)	Si (Atomic %)
Control (CPC)	-	37.45	62.55	-
5% SMPC	7.52	33.74	56.37	2.37
10% SMPC	7.78	31.93	57.81	2.48
20% SMPC	8.17	30.76	58.45	2.62

**Table 2:** Precursor components of CPCs and SMPCs.

Sample	TTCP (wt%)	DCPA (wt%)	TS (wt%)
Control (CPC)	72.91	27.09	─
5% SMPC	69.27	35.73	5.00
10% SMPC	65.62	24.38	10.00
20% SMPC	58.33	21.67	20.00

TTCP: tetracalcium phosphate; DCPA: dicalcium phosphate anhydrous; TS: tristrontium silicate.

## Data Availability

The data that support the findings of this study are available from the corresponding author upon reasonable request.
